# Practical Modeling of GNSS for Autonomous Vehicles in Urban Environments

**DOI:** 10.3390/s19194236

**Published:** 2019-09-29

**Authors:** Woosik Lee, Hyojoo Cho, Seungho Hyeong, Woojin Chung

**Affiliations:** School of Mechanical Engineering, Korea University, Seoul 02841, Korea; adhd@korea.ac.kr (W.L.); djowlrid@korea.ac.kr (H.C.); slkumquat@korea.ac.kr (S.H.)

**Keywords:** GNSS sensor model, odometry calibration, EKF localization

## Abstract

Autonomous navigation technology is used in various applications, such as agricultural robots and autonomous vehicles. The key technology for autonomous navigation is ego-motion estimation, which uses various sensors. Wheel encoders and global navigation satellite systems (GNSSs) are widely used in localization for autonomous vehicles, and there are a few quantitative strategies for handling the information obtained through their sensors. In many cases, the modeling of uncertainty and sensor fusion depends on the experience of the researchers. In this study, we address the problem of quantitatively modeling uncertainty in the accumulated GNSS and in wheel encoder data accumulated in anonymous urban environments, collected using vehicles. We also address the problem of utilizing that data in ego-motion estimation. There are seven factors that determine the magnitude of the uncertainty of a GNSS sensor. Because it is impossible to measure each of these factors, in this study, the uncertainty of the GNSS sensor is expressed through three variables, and the exact uncertainty is calculated. Using the proposed method, the uncertainty of the sensor is quantitatively modeled and robust localization is performed in a real environment. The approach is validated through experiments in urban environments.

## 1. Introduction

Recently, autonomous navigation technology has been used in various applications, such as agricultural robots, autonomous vehicles, and drones [1,2]. Various types of sensors are used to estimate position, for example, wheel encoders, global navigation satellite systems (GNSSs), inertial measurement units, cameras, and light detection and ranging (LiDAR). Among these sensors, wheel encoders and GNSS are the most widely used in autonomous navigation systems. Thus, constructing a precise model for wheel encoders and GNSSs can improve localization accuracy for autonomous vehicles. The models should be designed to consider both systematic and non-systematic errors.

The observation model and observation noise, which are parameters for modeling GNSSs, should consider systematic and non-systematic errors. Conventionally, systematic errors of the GNSS are not considered [3]; thus, the observation model is set as an identity element. The factors affecting non-systematic errors are ionospheric effects, tropospheric effects, ephemeris error, satellite clock error, multipath effects, and foliage attenuation. The effect of each factor is determined by the environment [4]. Thus, the observation noise should be set to effectively reflect these non-systematic errors. A typical method for establishing observation noise is to use the uncertainty information provided by sensors, and many studies have employed this information for localization [5,6,7]. However, both the actual positioning error and the error provided by the sensors are highly dependent on the performance of the GNSS sensor. Nevertheless, there are additional methods, such as applying neural networks. Afifi and El-Rabbany [8] proposed using the International GNSS Service-Multi-GNSS Experiment (IGS-MGEX) network to correct for satellite differential code biases and the orbital and satellite clock errors.

Thrapp, Westbrook, and Subramanian [9] proposed a method for determining the observation noise using the number of satellites. However, this method does not reflect individual non-systematic errors. Depending on the type of GNSS sensor, the area used, and the environmental conditions, the actual error and observation noise may not match. Wu, Jin, and Xia [10] proposed a GNSS sensor model that uses GNSS multipath errors, which vary depending on vegetation moisture content or the scatter size (stem or leaf) in a forest environment. Maier and Kleiner [11] and Taylor, Li, Brunsdon, and Ware [12] proposed a method for computing the uncertainty of the GNSS sensor, called the multipath effect. This method is based on calculating the uncertainty of the GNSS sensor for only those satellites with a signal path that is not obstructed. Although the method reduces the multipath effect and foliage attenuation, determination of the uncertainties of the remaining elements depends on the performance of the GNSS sensor. 

This study proposes using three variables to represent the seven non-systematic errors by categorizing the errors based on their characteristics. Of these three variables, two are determined via preliminary measurements, while the third variable is obtained by mapping according to the actual sensor error. This method can compute the observation noise independent of the performance of the sensor. Additionally, it is possible to construct a practical GNSS sensor model with a precision close to that of the sensors in urban environments. The main contribution of this study is applicable in most cases because it is the existing GNSS sensor that provides the magnitude of the uncertainty. However, in urban environments with many tall buildings, the multipath effect does not provide an accurate measure of the uncertainty. Therefore, this study models the uncertainty size of the GNSS sensor by measuring factors that contribute to its size. There are seven factors that determine the magnitude of the uncertainty of a GNSS sensor. Because it is impossible to measure each of these factors, in this study, the uncertainty of the GNSS sensor is expressed through three variables, and the exact uncertainty is calculated.

## 2. Integration of Odometry and GNSS

The improved odometry error model considers systematic and non-systematic errors. This section describes the systematic error of the odometry by straight and circular driving, and proposes a covariance matrix for non-systematic error modeling. 

### 2.1. Extended Kalman Filter (EKF)

The EKF allows the estimation of the state of a dynamic system given a sequence of observations and a control input. Observations originating from different sensors are typically combined to obtain a robust estimate of the state. Conventionally, the extended Kalman filter can be expressed as follows:

(1) $$x_{t}=g\left(u_{t-1},x_{t-1}\right)+\varepsilon_{t}$$

(2) $$z_{t}=h\left(x_{t}\right)+\delta_{t}$$

where $x_{t}$ is the state, $u_{t}$ is the control vector, and $z_{t}$ is the observation at time t. Here, g and h are the transition functions of state and observation, $\varepsilon_{t}$ and $\delta_{t}$ are zero-mean Gaussian noise variables with covariance $Q_{t}$ and $R_{t}$, and the EKF implements a linearization of g and h around the pose estimation computed in the previous time step t−1. Please refer to [13] for a more detailed description. 

### 2.2. The Conventional Odometry Motion Model

The control input model widely used in mobile robots is the odometry motion model. Two types of errors affect the accuracy of this odometry motion model. The first is a systematic error, which is a constant error that can be reduced by calibration. The second is a non-systematic error, where random errors occur, and the error can be reduced through statistical methods. Therefore, in this study, methods for reducing the systematic error and the non-systematic error of the existing odometry motion model are applied to an actual vehicle.

Jung [14] proposed a method that uses an oval track to accurately calibrate the odometry motion model based on the vehicle structure. For calibration, Jung suggests that the systematic errors comprise two representative variables: the wheelbase and the wheel diameter. Jung showed that the control input model can be effectively calibrated using the two representative variables by driving along the proposed oval track. However, the oval track includes both curved and straight paths. To perform control input model calibration experiments on this type of track, the vehicle must be modified to be controllable, with a very accurate steering angle control. Hence, it is difficult to apply this method to real vehicles.

### 2.3. Improved Odometry Motion Model for Vehicles

In this study, we propose a method for dividing the oval track into a straight track and a circular track, calculating the wheelbase and the wheel diameter of the vehicle by driving on the two tracks and calibrating the control input model simply. Because only one steering angle should be maintained on each track, control of the steering angle can be simplified, and no control modification is required.

**Figure 1:**
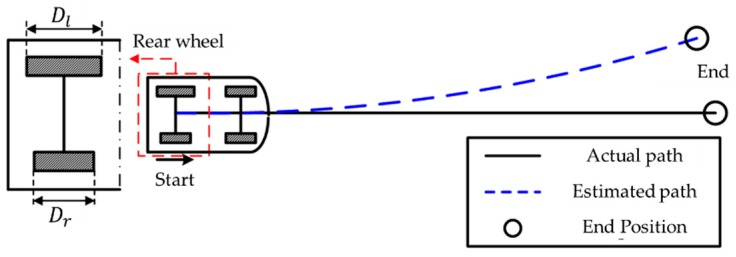
Comparing the actual path and odometry path with the wheel diameter error.

First, the wheel diameter error of the vehicle is calibrated by driving on a straight track. For a vehicle, driving straight is possible if the steering angle is maintained at zero. As indicated in Figure 1 by the black line, the actual travel distance of each rear wheel is the same as the actual travel distance of the car. However, if the diameters of the rear wheels are different, the measured distance on the odometer differs from the actual travel distance, as indicated in Figure 1 by the blue dotted line. If the actual travel distance L is known, both wheel diameters can be calibrated using a method from as:

(3) $$D_{\left(l,r\right)}=\frac{Lc_{\left(l,r\right)}}{\pi n_{\left(l,r\right)}}$$

where L is the actual travel distance of the car, $D_{\left(l,r\right)}$ is the actual left or right rear wheel diameter, $n_{\left(l,r\right)}$ is the estimated number of encoder pulses, and $c_{\left(l,r\right)}$ is the number of pulses per revolution of the encoders.

**Figure 2:**
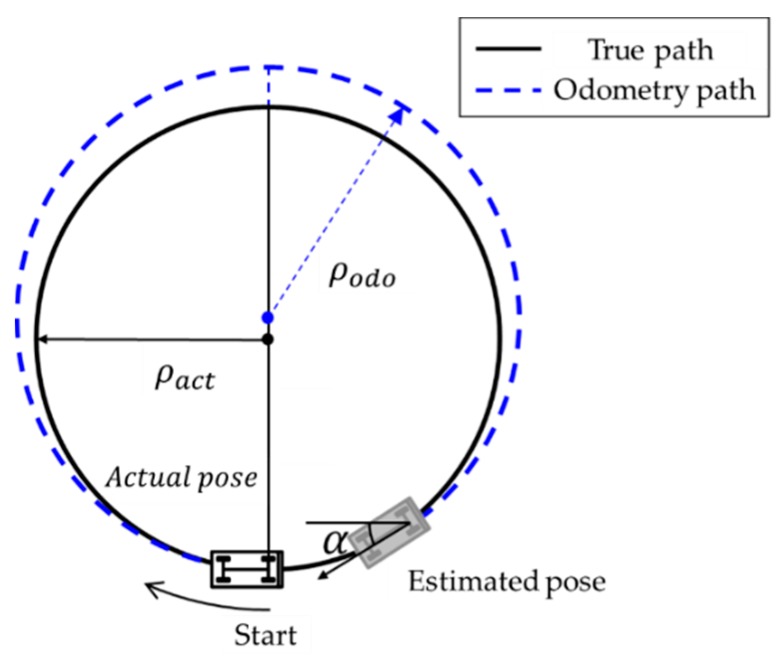
Comparing the actual path and odometry path with the wheelbase error.

Secondly, the wheelbase error of the vehicle is calibrated by driving along the circular track. If a constant steering angle is maintained, the car can follow a circular path with a radius of $\rho_{act}$, as shown in Figure 2, and the vehicle can return to its starting position. In this case, the measured odometry path appears as a blue path with a heading error of α and a radius $\rho_{odo}$. Using the formula for obtaining the heading angle from the encoder measurement pulse, the vehicle wheel track, $b_{cal}$, can be obtained, as shown in the following Equation (4):

(4) $$b_{cal}=b_{nom}\frac{\left(2\pi +\alpha \right)}{2\pi }$$

where $b_{cal}$ and $b_{nom}$ are the wheelbase after and before calibration, respectively.

Finally, we model the process noise of the vehicle odometry. Assuming the movements of both rear wheels are independent of each other, and the variance of the error is proportional to the travel distance, the non-systematic error covariance of the odometry can be set as follows [15]:

(5) $$\Sigma_{u_{t}}=\left[\begin{array}{cc} \sigma_{r,t}^{2} & 0\\ 0 & \sigma_{l,t}^{2} \end{array}\right]=\left[\begin{array}{cc} {\left(k_{r}{\;\Delta s}_{r,t}\right)}^{2} & 0\\ 0 & {\left(k_{l}{\;\Delta s}_{l,t}\right)}^{2} \end{array}\right]$$

where $\Sigma_{u_{t}}$ is the non-systematic error covariance of the odometry, $\Delta s_{l}$ and $\Delta s_{r}$ are the motion increments of the right and left wheels, and $k_{l}$ and $k_{r}$ are the non-systematic parameters of the travel distance of the right and left wheels, respectively. To calculate $\Sigma_{u_{t}}$, we measure $\Delta s_{l}$ and $\Delta s_{r}$ using the wheel encoder and set $k_{l}$ and $k_{r}$. We propose a method for determining $k_{l}$ and $k_{r}$ values using a straight track and circular track. First, the systematic error is removed using the control input model calibration, and the standard deviation of each wheel is calculated with a repetition of each straight and circular track. For localization stability, we use $k_{l}$ and $k_{r}$ as the larger values of standard deviation calculated from the straight and circular tracks. Using $k_{l}$ and $k_{r}$, we can calculate $\Sigma_{u_{t}}$ as the non-systematic error covariance and set the process noise. In actual vehicles, the movement of both wheels is independent due to the differential gear, therefore, it is applied to the extended Kalman filter through the equation used in the motion model for the two-wheeled robot. The detailed equation of the motion model is as follows [15].

(6) $$p_{t+1}=g\left(x,y,\theta ,{\Delta s}_{r},{\Delta s}_{l}\right)=\;\left[\begin{array}{c} x+\frac{\Delta s}{2}\cos\left(\theta +\frac{\Delta \theta }{2}\right)\\ y+\frac{\Delta s}{2}\sin\left(\theta +\frac{\Delta \theta }{2}\right)\\ \Delta \theta \end{array}\right]$$

(7) $$\Sigma_{p_{t+1}}=\nabla_{p_{t}}g\cdot \Sigma_{p_{t}}\cdot \nabla_{p_{t}}g^{T}+\nabla_{\Delta_{rl}}g\cdot \Sigma_{u_{t}}\cdot \nabla_{\Delta_{rl}}g^{T}$$

where $p_{t}$ represents the position at time t and is calculated as in Equation (6). Here, $\Sigma_{p_{t}}$ is the covariance of the motion model at time t and is calculated as shown in Equation (7) using the propagation of the error.

Therefore, using the proposed straight and circular tracks, it is possible to set the control input model and process noise of the odometry using the encoder installed in the actual car, and consequently, the odometry motion model can be constructed practically.

## 3. Improved GNSS Sensor Model

The GNSS sensor model is the $z_{t}=h\left(x_{t}\right)+\delta_{t}$ portion of Equation (2). The observation noise $\delta_{t}$ should be specified. To match the observation noise to the actual operating characteristics of the GNSS sensor, non-systematic errors should be considered without using the uncertainty information provided by the sensor.

The factors affecting the non-systematic errors of the GNSS sensor are the delusion of precision (DOP) and ranging errors [4]. Ranging errors can be further classified into receiver noise, ionospheric effects, atmospheric effects, ephemeris errors, satellite clock errors, multipath effects, and foliage attenuation. The uncertainty of the GNSS sensor is calculated as follows [4]:

(8) $$\sigma_{G}=DOP\times \sqrt{\sigma_{I}{}^{2}+\sigma_{T}{}^{2}+\sigma_{E}{}^{2}+\sigma_{S}{}^{2}+\sigma_{M}{}^{2}+\sigma_{F}{}^{2}+\sigma_{R}{}^{2}}$$

where $\sigma_{G}$ represents the uncertainty of the GNSS sensor; $\sigma_{I}$, $\sigma_{T}$, $\sigma_{E}$, $\sigma_{S}$, $\sigma_{M}$, $\sigma_{F}$, and $\sigma_{R}$ represent the magnitudes of uncertainty due to ionosphere influences, atmospheric effects, ephemeris errors, satellite clock errors, receiver error, multipath effects, and foliage attenuation, respectively. The DOP is not related to the position estimation performance of the GNSS sensor because it is determined by the geometrical arrangement of the satellite. Therefore, the DOP received by the GNSS sensor is not significantly different from the actual DOP. In contrast, the seven error factors included in the ranging errors are directly related to the position estimation performance of the GNSS sensor. Thus, if the uncertainty of each factor can be accurately measured, the observation noise can be accurately calculated based on the characteristics of the GNSS sensor. However, measuring the uncertainty of the seven error factors is impractical with regard to system construction cost and efficiency.

Therefore, we propose a method for constructing the GNSS sensor model by expressing the seven error factors as three representative variables and setting the observation noise by calculating the value of each representative variable through three experiments.

We represent the seven error factors with three representative variables based on the characteristics of each error: receiver error (RE), atmospheric effect and satellite-oriented error (ASE), and local characteristic error (LCE). The relationship between each representative variable and the error factors is as follows:

(9) $$\mathrm{RE}=\sigma_{R}$$

(10) $$\mathrm{ASE}\;\left(\mathrm{model}\right)=\sqrt{\sigma_{I}{}^{2}+\sigma_{T}{}^{2}+\sigma_{E}{}^{2}+\sigma_{s}{}^{2}}$$

(11) $$\mathrm{LCE}\;\left(\mathrm{position}\right)=\;\sqrt{\sigma_{M}{}^{2}+\sigma_{F}{}^{2}}$$

The RE depends on uncertainty due to receiver noise. ASE depends on ionospheric influence, atmospheric effect, ephemeris error, and uncertainty due to satellite clock error. These four error factors are characterized by the magnitude of the uncertainty, which can be determined using the calibration model used in the measurement of the GNSS sensor. Notably, the real-time kinematic (RTK) reduces the ASE to a negligible value [5]. Therefore, in this study, the square root of the sum of squares of these four errors is expressed as 𝐴𝑆𝐸 (model), based on the type of calibration model. Finally, the LCE represents the sum of uncertainties due to multipath effects and foliage attenuation. Because these two error factors are heavily influenced by the measurement environment, modeling is difficult. Therefore, in this study, the 𝐿𝐶𝐸 (𝑝𝑜𝑠𝑖𝑡𝑖𝑜𝑛) is constructed by mapping the sum of the uncertainties from the two factors based on the measurement location and creating a local characteristic error map. Equations (9)–(11) are combined with Equation (12) when defining the relationship between the uncertainty of the GNSS sensor and the three representative variables.

(12) $$\sigma_{G}=DOP\times \sqrt{ASE{\left(state\right)}^{2}+LCE{\left(position\right)}^{2}+RE^{2}}$$

The experimental method for measuring each variable is as follows: First, the RE is calculated from measurements in an environment where the ASE and LCE are assumed to be zero. When the RTK solution is used as a calibration model, the ASE is assumed to be 0 [16]. The LCE is assumed to be 0 if the measurement environment is the roof of a building or an open space where multipath effect and foliage attenuation do not occur. Then, $\sigma_{G}$ is calculated from the measurement from the fixed GNSS sensor at a certain time. The DOP uses the value provided by the sensor. We calculate the RE value using Equation (9). Second, we measure the ASE value in an environment where the LCE value is assumed to be 0. Here, $\sigma_{G}$ and the DOP are measured from a fixed position relative to the GNSS sensor, as in the previous experiment. The ASE is calculated via Equation (12) using the measured $\sigma_{G}$ and DOP and the pre-calculated RE value obtained using calibration models. Third, the LCE values are measured using the pre-computed RE and ASE values in an environment where the GNSS sensor model is to be applied. The variable LCE is calculated through repeated driving, and the calculated LCE value is stored by dividing the area by a certain distance. Details are given in Section 4.3.3. The $\sigma_{G}$ value is calculated by repeatedly measuring the same environment. By using the measured DOP and pre-calculated RE and ASE values, the local characteristic error map is generated using the LCE values measured for each region via Equation (12). The measured GNSS sensor data is presented in Cartesian coordinates using the WGS84 method. The detailed equation of the GNSS sensor model is as follows.

(13) $$R_{t}=\left[\begin{array}{cc} {\sigma^{\prime }}_{G_{t}} & 0\\ 0 & {\sigma^{\prime }}_{G_{t}} \end{array}\right]$$

where the ${\sigma^{\prime }}_{G}$ calculated by substituting ASE, LCE, and RE variables into Equation (12) is used to update the position.

The advantage of this method is that it reduces the number of experiments needed to construct a GNSS sensor model. Furthermore, the uncertainty of the position measurement can be calculated without using the uncertainty provided by the GNSS sensor. Thus, the GNSS sensor model can be accurately constructed.

## 4. Experimental Results

### 4.1. Experimental Setup

**Figure 3:**
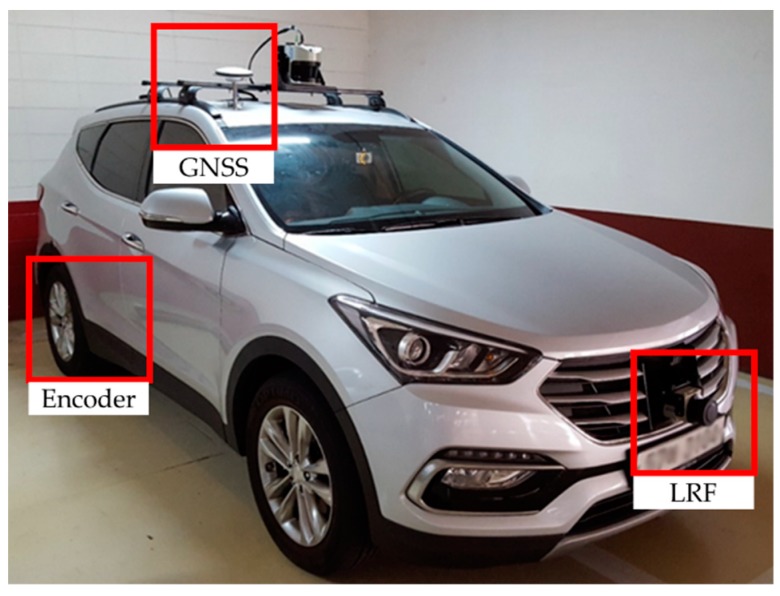
Experimental platform: Santa Fe DM 2.0 2WD with an Autonics E40HB wheel encoder, Novatel Propak-V3, and SICK LMS111. Note: GNSS = global navigation satellite system; LRF = laser range finder.

Figure 3 shows the car platform used in this study. The platform is based on a Santa Fe DM 2.0 2WD. An Autonics E40HB wheel encoder was installed on both sides of the rear wheel. A GNSS (NovAtel Propak-V3) was mounted on the roof of the vehicle above the center of the rear axle of the car. Furthermore, a laser rangefinder (SICK LMS111) was installed on the front of the vehicle with a vertical orientation to the ground. We used the scan data measured by the laser rangefinder to estimate the vehicle position and as ground truth data.

### 4.2. Motion Model Construction

To construct the wheel odometry motion model, the experimental environment was set to reflect an actual road. We obtained the ground truth of the vehicle using a NovAtel Propak-V3, with the calibration model as the RTK. In the experiment, ground truth precision was 0.02 m (1σ).

#### 4.2.1. Calibration of Wheel Diameter Through Straight Driving

**Figure 4:**
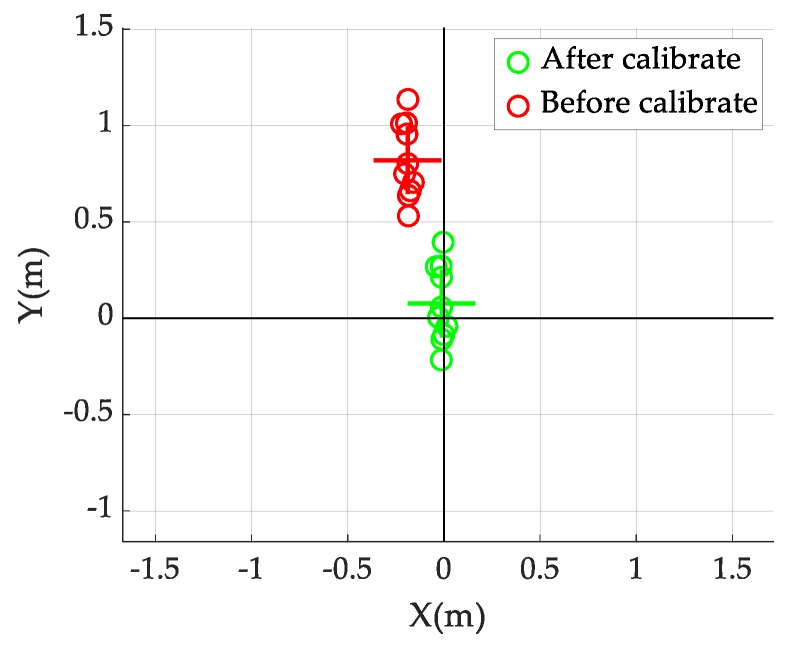
Comparison of the straight driving paths before and after wheel diameter error calibration.

A straight driving test was performed in a parking lot to calibrate the wheel diameter error of the car. The straight driving test was performed 10 times along a 50 m straight path (L), as shown in Figure 4. To minimize the effect of non-systematic errors caused by vehicle dynamics, the vehicle was driven at a speed of ≤30 km/h. $D_{r}$ and $D_{l}$ were the encoder measurements from each wheel. The calibrated diameters were calculated to be $D_{r}$ = 721.6 mm and $D_{l}$ = 722.3 mm from the mean value of the results obtained after 10 runs.

Figure 4 shows the error of the 50 m straight driving test before and after calibration of the wheel diameter error. The red and green symbols represent the odometry error before and after calibration, respectively. After utilizing this calibration technique, the odometry error was reduced. The average final position error after calibration was 0.077 m, which is approximately 10.9 times better than the final position error before calibration (0.841 m). Thus, $D_{r}$ and $D_{l}$ were properly calibrated using the proposed scheme.

#### 4.2.2. Wheelbase Calibration Through Circular Driving

Once the wheel diameter error of the vehicle was corrected, the systematic odometry error in a circular driving test was used as the wheel track error. The circular driving test was performed five times in clockwise (CW) and counterclockwise (CCW) directions while keeping the steering angle constant. 

**Figure 5:**
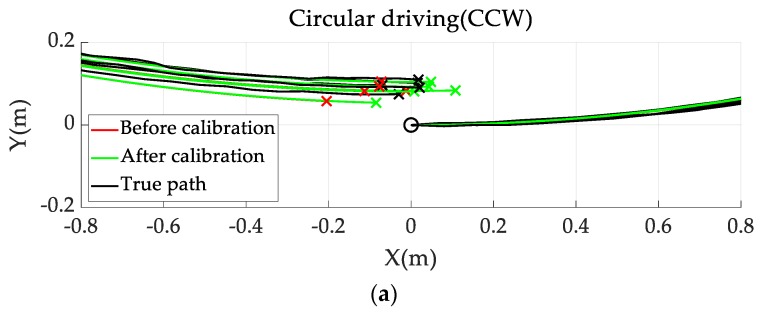

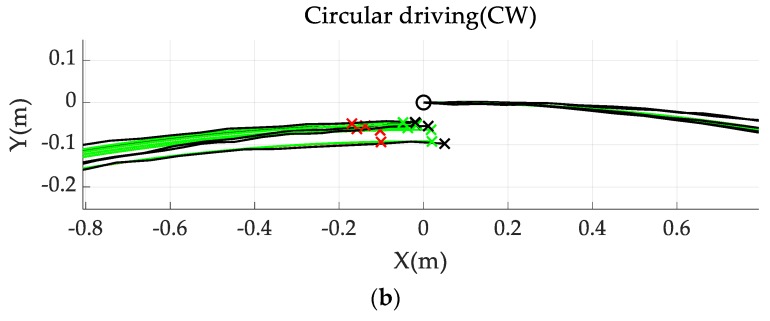
Result for the circular path before and after calibration. Comparison of circular path results: (**a**) counterclockwise (CCW); (**b**) clockwise (CW).

Figure 5 shows the results of the circular driving test before and after wheel track calibration. The squares and circles represent the circular paths of the vehicle before and after calibration, respectively. The heading angle odometry error before calibration was α𝑐𝑐𝑤 = −1.3° in the CCW direction and α𝑐𝑤 = −1.5° in the CW direction. Here, 𝑏𝑐𝑎𝑙𝑖 was calculated as 1632.8 mm from the measured α. As shown in Figure 3, after calibration, the odometry error was reduced. Furthermore, the final position calculated via odometry was corrected to close to zero. The final position error before calibration was distributed at a position distant from the origin. The average final position error after calibration was 0.035 m in the CCW direction and 0.015 m in the CW direction, which is 2.5 and 9.0 times superior to the pre-calibration values of 0.035 m in the CCW direction and 0.135 m in the CW direction, respectively. Thus, the wheel track was properly calibrated using the proposed scheme.

#### 4.2.3. Non-Systematic Error Parameter Estimation

The control input model was constructed using the corrected wheel diameters and wheelbase, and the non-systematic parameters ($k_{l}$, $k_{r}$) of each wheel were calculated. The previously obtained straight track driving and circular track driving data were used in the calculation of the process noise parameters. Table 1 presents the non-systematic parameters calculated for each track. Because the non-systematic parameter is larger for the circular track than for the straight track, we selected $k_{l}$ = 0.0279 and $k_{r}$ = 0.0292 as the process noise parameters of the circular track for the construction of the sensor model.

#### 4.2.4. Motion Model Verification Experiment

**Figure 6:**
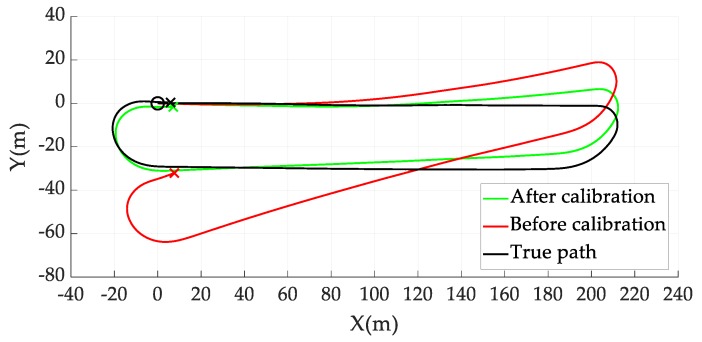
Comparison of the circular driving paths before and after wheel diameter and wheelbase calibration.

Experiments were conducted to verify the wheel odometry motion model. Encoder data was collected while driving on the oval track for approximately 200 m. The results of the wheel odometry motion model verification are presented in Figure 6. The post-calibration path has a similar shape to the actual path (compared with the pre-calibration path), and the final position error is reduced. The final position error for the odometry path was calculated to be 32.79 m before calibration and 2.44 m after calibration. The mean error of the total path was 20.99 m before calibration and 4.61 m after calibration. Therefore, the systematic error was reduced via the control input model calibration using the proposed method.

### 4.3. Sensor Model Construction

For construction of the GNSS sensor model, the RE and ASE were measured in environments where the multipath effect and foliage attenuation were negligible, as shown in Figure 8. We used the NovAtel Propak-V3 as the GNSS sensor and measured sensor data at 10 Hz for 10 min in three different environments. The measured data were used to calculate the RE and ASE. Three types of calibration models were used: integer RTK, float solution, and no data (no calibration model). In this experiment, we used a horizontal DOP because it captures the influence of the satellite constellation on the position estimate in the horizontal plane, while ignoring the vertical component, given that the vehicle commonly drives in a horizontal plane. The measured results are presented in Table 2. The values of the RE and ASE were set as the mean values in each environment.

#### 4.3.1. RE Measurement

We assumed that the ASE value was zero and used integer RTK as the calibration model. Then, the RE value of the NovAtel Propak-V3 was calculated to be 0.02 m.

#### 4.3.2. ASE Measurement

Table 2 presents the calculated ASE (state) for each calibration model. The calibration models (except for the integer RTK where the uncertainty was assumed to be zero) were calculated to have uncertainty that was dependent on the state. The networked transport of radio technical commission for maritime services (RTCM) via internet protocol (NTRIP) method was used for the GNSS RTK [17]. NTRIP is a technique for calibrating an existing GNSS model by receiving calibration signals from a network. The difference between float and integer solutions is in the format of ambiguity resolution. Integer RTK resolution at a single station can be achieved by introducing predetermined, uncalibrated phase delays into the float ambiguity estimates of precise point positioning [18].

#### 4.3.3. LCE Measurement

**Figure 7:**
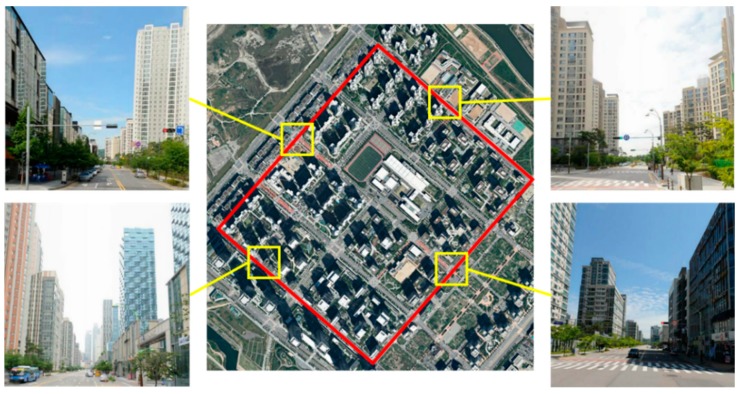
Driving environment in an urban environment with multipath effect that interferes with GNSS satellite signals due to high buildings.

**Figure 8:**
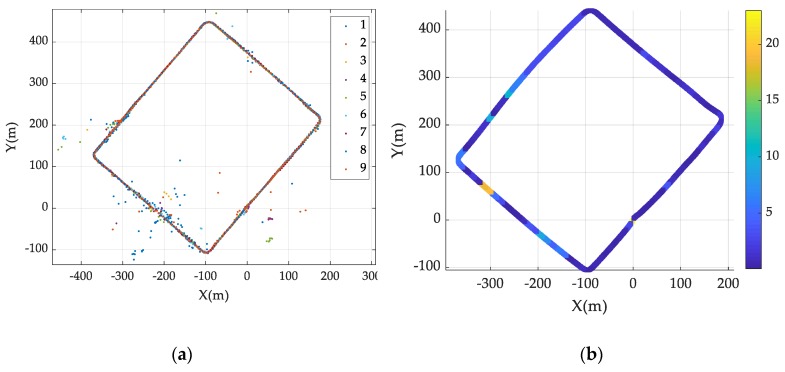
(**a**) GNSS measurement data measured in 9 driving tasks. (**b**) Results of the local characteristic error map generation.

The experimental environment for the LSE value was a 2.4 km city road, as shown in Figure 7. The experiment was performed by driving along the yellow line. The car started at the red point and stopped at the blue point. We obtained the GNSS sensor data by driving 10 times, and the vehicle speed did not exceed 50 km/h. The experimental environment can be classified into two types based on environmental characteristics. The first environment had a large multipath effect and foliage attenuation due to buildings, as shown in Figure 7. The ground truth for calculating the GNSS position error was obtained using the SICK LMS111 mounted on the front of the vehicle, as well as odometry. The horizontal standard deviation error of the ground truth was 0.2 m (1σ). The results of the GNSS sensor measurement during nine driving tasks are presented in Figure 8a. The LCE variable is calculated from the GNSS data collected by driving the experiment site nine times. With the precalculated RE and ASE values, and the measured DOP and $\sigma_{G}$, we derive the LCE, as shown in Equation (10). The calculated LCE map is shown in Figure 8b.

#### 4.3.4. GNSS Sensor Model Verification

**Figure 9:**
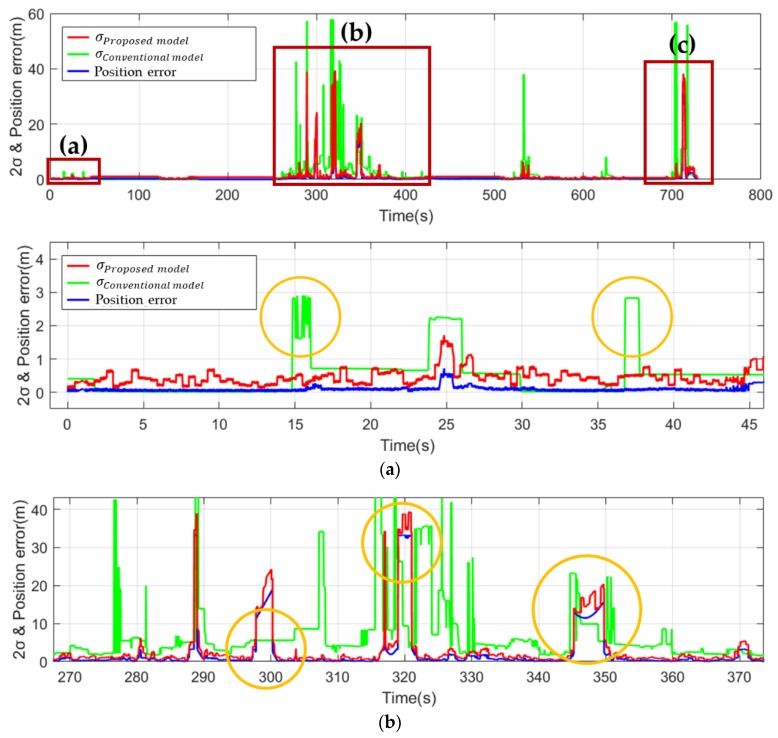

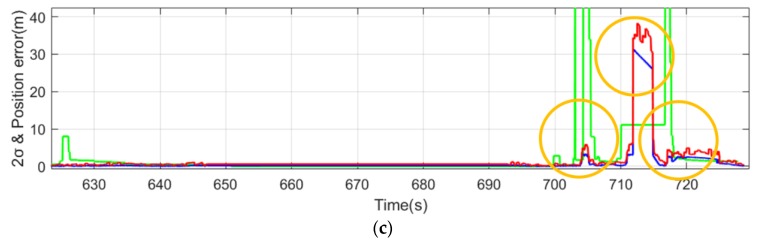
Comparison of the estimated error between the proposed method (blue line) and the conventional method (green line).

To verify the GNSS sensor model constructed using the RE, ASE, and LSE, we compared the uncertainties of the sensor and the proposed GNSS sensor model. The numbers of ground truths included in the error ellipses of different confidence intervals were compared, and the results are presented in Figure 9. In Figure 9, the blue line denotes position error, the green line is twice the uncertainty provided by a conventional sensor model, and the red line is twice the uncertainty of the proposed method. There is no significant change in the section where the GNSS sensor operates well. However, where the signal blindness of the GNSS sensor increases, the proposed technique accurately represents the uncertainty of the sensor. 

We compare the results from the conventional GPS sensor model with the results from the improved sensor model [19]. The RMS between GNSS measurement error and estimated error (2σ) over the entire interval of the experiment is 0.42 m in the proposed model and 1.08 m in the conventional method, as shown in Table 3. This means that the proposed uncertainty model accurately represents the uncertainty of the actual sensor. The number of epochs in the 2σ ellipse range should theoretically be 95%, it should be 97.6% for the proposed model, and 72.8% for the conventional model. The proposed method accurately estimates the theoretical 2σ range. Figure 9 compares the magnitude of the uncertainty of the proposed GNSS sensor model with that of the conventional GNSS sensor model. The closer the estimated uncertainty is to the actual position error, the higher the accuracy of the sensor model. The environment in Figure 9a is an environment with few high buildings around, and the environment is more certain due to the multipath effect. The environment in Figure 9b,c is high in uncertainty caused by the GNSS sensor because of the surrounding high buildings and trees. In the conventional method, the uncertainty magnitude of the GNSS sensor is predicted to be larger or smaller than the actual position error. However, the proposed method has a value that is similar to the position error.

### 4.4. EKF Localization Using the Proposed Models

**Figure 10:**
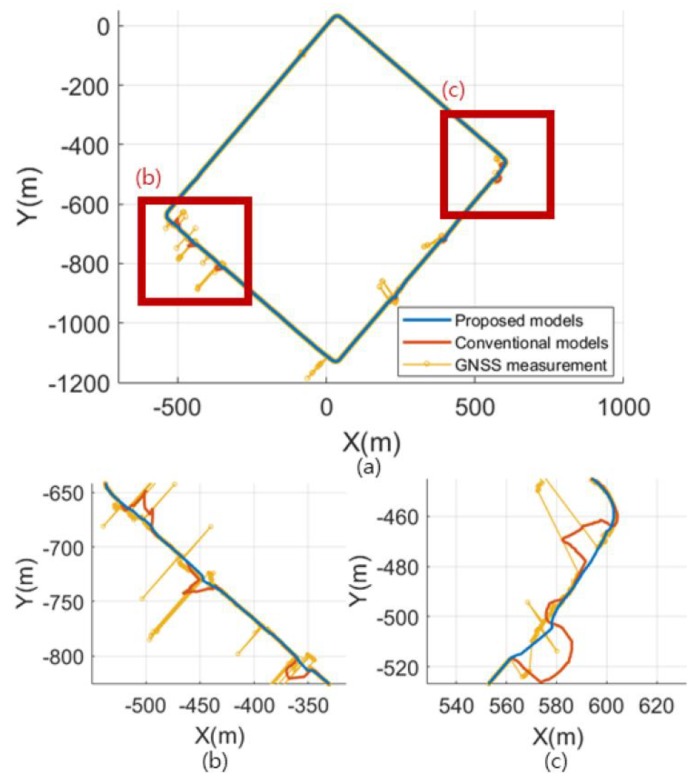
Comparison of the Extended Kalman filter (EKF) localization results of the proposed and conventional methods.

To evaluate the effectiveness of the proposed model, we compared the EKF localization results obtained using the proposed and conventional models. This experiment was performed on hardware with a Core i5-6600 3.30GHz. The code was written using MATLAB. EKF localization using the proposed and conventional models was performed at 10 positions. Figure 10 shows the results for experiment place 3. If the GNSS sensor measurement is accurate, there will be no significant difference between the proposed model and the conventional model. However, Figure 10b shows a result where the GNSS sensor measurement was inaccurate, and the EKF localization performance of the proposed model was more stable than that of the conventional model. Similarly, in Figure 10c, the conventional method models the uncertainty of the GNSS sensor smaller than it is, resulting in a large error. Table 4 shows the results of EKF location estimation using the proposed technique in different experimental environments. The position error is reduced in all experimental environments. Environment 1 in Table 4, where the surrounding buildings are high, shows a high-performance improvement.

## 5. Conclusions

We proposed a practical method for constructing an odometry motion model and a GNSS sensor model. The odometry motion model was constructed using a circular track and a straight track, and the model constructed using the oval track driving results was verified. The GNSS sensor model was composed of ground truth contained in the error ellipse between the proposed model and the model provided by the sensor. Finally, we compared the results of EKF location estimation using the proposed motion model and the sensor model to the EKF location estimation results obtained using the pre-correction motion model and the GNSS sensor-provided model for three positions. The proposed models exhibited higher estimation accuracy. In all experiments, the position error is improved by 46% on average. Good performance is also shown for environments with tall buildings and trees. In future work, we will consider extending the approach towards a local characteristic error map that is updated in real time.

## Figures and Tables

**Table 1:** Estimation results for $k_{l}$ and $k_{r}$ (m/m).

	Test Run		
	Straight	CCW	CW
$k_{l}$	0.0047	0.0279	0.0141
$k_{r}$	0.0048	0.0176	0.0292

**Table 2:** Calculated ASE values for each calibration model.

State	ASE (state)
None	0.89 m
Float Solution	0.69 m
Integer RTK	0.00 m

**Table 3:** Result of GNSS sensor model verification.

	RMS between Measurement Error and Estimated Error (2σ)	Number of Epochs in the 2σ Ellipse
Proposed	0.42 m	97.6%
Conventional	1.08 m	72.8%

**Table 4:** Error for position estimation (m).

Error	Conventional Model	Proposed Model	Difference
	Experiment place 1		
Position			
Avg.	28.5 m	4.9 m	96.50%
Max.	56.7 m	8.5 m	
	Experiment place 2		
Position			
Avg.	4.4 m	3.6 m	18.20%
Max.	15.4 m	10.6 m	
	Experiment place 3		
Position			
Avg.	7.0 m	5.3 m	24.30%
Max.	56.2 m	14.0 m	

## Abbreviations

GNSS	Global navigation space system
RTK	Real-time kinematic
NTRIP	Networked transport of RTCM via internet protocol
LIDAR	Light detection and ranging
RE	Ranging error
ASE	Atmospheric effect and Satellite-oriented error
LCE	Local characteristic error
